# Molecular Evolution of Influenza A Viruses From Mauritius, 2017–2019

**DOI:** 10.1111/irv.70108

**Published:** 2025-05-13

**Authors:** Magalutcheemee Ramuth, Janaki Sonoo, Fhooblall Mokshanand, Belinda Herring, Tessema Sofonias, John McCauley, Ashwamed Dinasing, Florette K. Treurnicht

**Affiliations:** ^1^ Central Health Laboratory, Victoria Hospital, Ministry of Health & Wellness Quatre‐Bornes Plaine‐Wilhems Mauritius; ^2^ Division of Medical Virology, School of Pathology, Faculty of Health Sciences University of Witwatersrand Johannesburg South Africa; ^3^ World Health Organization, Regional Office for Africa Brazzaville Republic of the Congo; ^4^ Africa Centers for Disease Control and Prevention (Africa CDC) Addis Ababa Ethiopia; ^5^ Worldwide Influenza Centre The Francis Crick Institute London England; ^6^ Ministry of Health & Wellness Port Louis Plaine‐Wilhems Mauritius; ^7^ Department of Virology National Health Laboratory Service Parktown Johannesburg South Africa

**Keywords:** Ct cycle threshold, GISAID Global Initiative on Sharing All Influenza Data, HA Haemagglutinin, ILI Influenza Like Illness, NA Neuraminidase, NGlyc N‐linked glycosylation, PCR Polymerase chain reaction, SARI Severe Acute Respiratory Illness

## Abstract

**Background:**

Despite being a vaccine preventable disease, influenza remains a burden in African countries. In Mauritius, influenza virus activity is year‐round but evidence‐based data to guide vaccination and pandemic preparedness strategies are lacking. This study aimed to describe the genetic diversity of influenza A viruses detected in Mauritius between 2017 and 2019.

**Methods:**

Influenza A/H1N1pdm09 and A/H3N2 virus isolates were sequenced using Oxford Nanopore technology. Sequence reads assembled by CZ ID and Genome Detective web‐based tools were uploaded to the EpiFlu database of the Global Initiative on Sharing All Influenza Data (GISAID). Sequence alignments and phylogenetic analysis were performed using Nextclade and MEGA XI software. BioEdit software was used to view amino acid substitutions compared to annual vaccine strains. Prediction of potential N‐linked glycosylation (PNG) sites was determined by NetNGlyc 1.0.

**Results:**

Influenza A was predominant (92.6%), with A/H1N1pdm09 prevailing overall (62.5%) but A/H3N2 dominating in 2017 (55.9%). Phylogenetic analysis identified clade 6B dominance for A/H1N1pdm09, with notable substitutions E119K, Q136K and D151E linked to antigenic changes. A/H3N2 exhibited significant genetic diversity, with co‐circulation of 3C.2a4 and 3C.2a1 in 2017 while 2018 predominant subclade 3C.2a1b.1 highlights continued antigenic drift. Loss of PNG sites at position 158 (11/21; 52.4%) in HA and position 329 (81.0%, 17/21) in NA of A/H3N2 viruses were observed.

**Conclusions:**

Continued evolution of A/H1N1pdm09 and A/H3N2 viruses in Mauritius highlights the need for sustained genomic surveillance to inform vaccine and antiviral strategies. Data from Mauritius will contribute to understanding of influenza viruses' ecology in the African region and globally.

## 1 Introduction

Influenza viruses belong to the Orthomyxoviridae family and consist of eight negative‐sense RNA segments: PB2, PB1, PA, HA, NP, NA, M, and NS in order of decreasing length within an enveloped virion and classification is guided by the International Committee on Taxonomy of Viruses (ICTV) [1]. Influenza A viruses cause annual epidemics and unpredictable pandemics, with ones in 1918 (H1N1), 1957 (H2N2), 1968 (H3N2), and 2009 (H1N1) [2] being best studied. Genetic mutation leading to antigenic drift and gene reassortment leading to antigenic shift are considered some of the most important molecular mechanisms in the evolution of influenza A virus and the World Health Organization's Global Influenza Surveillance and Response System (WHO‐GISRS) [3] enables an effective early warning system for public health actions.

Mauritius, a tropical island with a population of 1.24 million as at July 2024 [4] has a history of influenza dating back to the Spanish flu pandemic in 1919 [5] and experiences influenza year‐round with peaks in summer and winter. Since 2009, the government has provided free influenza vaccines to vulnerable groups such as the elderly, children under five, pregnant women, healthcare workers, and individuals with chronic medical conditions.

Tropical African countries are advised to administer the most recent vaccine formulation; however, occasionally due to antigenic drift of circulating strains or incorporation of egg‐adaptive substitutions during egg‐based vaccine production, vaccine mismatch occurs leading to an impact on vaccine effectiveness [6, 7, 8]. The choice to select between Northern or Southern vaccine formulations is critical for advocating health policies and can only be achieved if country specific scientific evidence supports it [9].

Next‐generation sequencing (NGS) has become crucial for influenza virus surveillance, predicting susceptibility to antivirals, and tracking novel strains [10, 11]. Nanopore sequencing, particularly through devices like the MinION MK1c, has emerged as a valuable tool for real‐time genomic surveillance. During the coronavirus disease of 2019 (COVID‐19) pandemic, African countries, including Mauritius, adopted this technology to monitor pathogens and are now expanding its use for other viruses [12].

## 2 Materials and Methods

### 2.1 Study Setting and Study Population

Influenza virus surveillance was conducted at five sentinel sites, namely Victoria Hospital, Dr. Jeetoo Hospital, Sir Seewoosagur Ramgoolam National hospital, Jawaharlal Nehru hospital, Flacq (SAJ) hospital and a network of regional and community health centers throughout Mauritius. The study included in‐ and out‐patients of all ages with severe acute respiratory illness (SARI) and influenza‐like illness (ILI) respectively, during 2017 to 2019.

### 2.2 Ethics

The study protocol approved by Mauritius Ministry of Health and Wellness ethics committee MHC/CT/NETH/RAMMA and the University of Witwatersrand Human Research Ethics committee (M2010/17 MED20‐08‐208) Johannesburg, South Africa.

### 2.3 Study Samples

Throat and nasopharyngeal swabs were collected in viral transport medium and transported to the Mauritius National Laboratory within 24 h. Samples were divided into three aliquots for RT‐PCR, virus isolation, and archiving at −70°C.

### 2.4 Influenza Virus Diagnosis by Real‐Time RT‐PCR

Collected samples were initially screened for influenza A/B viruses using the Invitrogen Superscript™ III Platinum® One‐Step qRT‐PCR reagent kit (Thermo Fisher Scientific, Invitrogen, USA) and protocol as described [13]. Similarly, influenza A virus subtyping was also done using Centers for Disease Control and Prevention (CDC) real‐time reverse transcription‐based polymerase chain reaction (RT‐PCR) assays targeting the haemagglutinin (HA) genes of influenza A viruses. A sample was considered positive if results from two different RT‐PCR targets (universal M gene and HA gene) were both positive (cycle threshold [Ct] values ≤ 38.0).

### 2.5 Influenza Virus Isolation

Madin‐Darby Canine Kidney (MDCK) cell monolayers in culture tubes were maintained in medium 199 containing Earle's salts and L‐glutamine (Merck KGaA, Darmstadt, Germany) supplemented with 50–100 IU/mL penicillin, 50–100 μg/mL streptomycin, 2.2 g/L sodium bicarbonate and 2% FBS. MDCK cells were inoculated with 400 μL of each sample positive for influenza A viruses. Following absorption of virus inoculum for 30 min at 35°C, medium 199 supplemented with 10% (v/v) bovine serum albumin (fraction V; Sigma Aldrich, Darmstadt, Germany) were added and cultures were incubated at 35°C for and examined daily for cytopathic effect up to 10 days. Successful viral cultures were confirmed by haemagglutination titres using Human O Red blood cells [14] of supernatants harvested.

### 2.6 Influenza A Virus Genome Amplification

RNA was extracted from virus isolates using the QIAamp Viral RNA Mini kit (Qiagen, Hilden, Germany). Universal influenza A primers were used to amplify all genome segments in a single reverse‐transcription‐based PCR (RT‐PCR) [15]. Reverse transcription was done at 42°C for 15 min (min), 55°C for 15 min, and 60°C for 5 min and hold at 94°C for 2 min. Thermocycling followed with 5 cycles at 94°C for 20 s, 58°C for 30 s and 68°C for 3 min; thereafter 40 cycles at 94°C for 20 s, 58°C for 30 s and 68°C for 3 min; and final extension at 68°C for 10 min [16]. PCR products were visualized on an ultraviolet transilluminator following electrophoresis through a 1.5% agarose gel and staining with 0.6 μg/mL of ethidium bromide (Invitrogen, Life Technologies, Carlsbad, California, USA) [17].

### 2.7 Nanopore Sequencing of Influenza A Viruses

Amplicons were quantified with the Qubit™ 1× dsDNA High Sensitivity (HS) Assay (Invitrogen, Life Technologies, Carlsbad, California, USA) and normalized to 80 ng/μL. Normalized DNA samples were barcoded using the Native barcodes 1–96 (Oxford Nanopore Technologies, Oxford, UK). Barcoded samples were pooled (up to 96 samples per flow cell) and DNA libraries were treated with 1× AMPure XP beads to remove unbound barcodes and primers. Rapid adapter was added to 600–800 ng of each library, mixed and incubated for 5 min before it was loaded into a primed FLO‐MIN106 R9.4.1 Flow Cell (Oxford Nanopore Technologies, Oxford, UK). The MinION MK1c device was set to run for 48 h using high accuracy base calling in the MinKNOW.

### 2.8 Analysis of Nanopore MinION MK1C Sequence Reads

Base calling and demultiplexing of the fast5 files were performed using the Oxford Nanopore integrated Guppy software version 6.3.9 (https://github.com/articnetwork/rampart). Base called reads in FastQ format and a minimum error rate of 8% was used to filter reads that passed quality control to obtain an expected base call accuracy of 92% or an expected average Phred quality score of 15 to 20. The FastQ files were assembled using the CZ ID analysis platform (https://czid.org) as well as the Genome Detective Tool version 2.52 (https://www.genomedetective.com/app/typingtool/virus/).

### 2.9 Phylogenetic Analyses

Consensus sequences generated from influenza A/H1N1pdm09 and A/H3N2 virus gene segments with HA and overall genome coverage ≥ 80% were submitted to the EpiFlu database of the Global Initiative on Sharing All Influenza Data (GISAID) (https://gisaid.org/). The Nextclade analysis tool was used for multiple sequence alignment and to assign clade or lineages and sublineages (https://nextstrain.org/). Amino acid mutational analysis was done using BioEdit [18] and Notepad ++ (notepad‐plus‐plus.org).

Evolutionary history for influenza A viruses' HA and NA gene sequences was inferred by using the Maximum Likelihood method with Tamura‐Nei model in Molecular Evolutionary Genetics Analysis MEGA software version 11 [19] with 1000 bootstraps to assess reliability. Bootstrap values above 70% were indicated on branches. The Figtree tool was used for graphic visualization and editing of the generated Newick format tree [20].

All the HA and NA gene sequences of A/H1N1pdm09 and A(H3N2) vaccine strains were obtained from the GISAID EpiFlu database. For influenza A/H1N1pdm09, the A/Michigan/45/2015 (EPI_ISL_199532) cell culture‐based sequence was used as reference (Table S1). The representative HA and NA gene sequences of A(H3N2) egg propagated virus for 2017, 2018, and 2019 were A/Hong Kong/4801/2014 (EPI_ISL_16713985), A/Singapore/INFIMH‐16‐0019/2016 (EPI_ISL_239803), and A/Switzerland/8060/2017 (EPI_ISL_319720), respectively. Other reference influenza A virus sequences were also retrieved from GISAID (Table S2c and d).

### 2.10 Mutational Analysis of HA and NA Gene Segments

Mutational analysis of the deduced amino acid sequences of the Mauritian influenza A/H1N1pdm09 and A/H3N2 viruses' haemagglutinin (HA), neuraminidase (NA) proteins, matrix (M1 and M2) protein, and C‐terminal domain of polymerase (PA) protein [21] was done using FluSurver (https://flusurver.bii.a‐star.edu.sg/). H1 numbering (excluding signal peptide) was used for A/H1N1 viruses sequence analysis and H3 numbering (excluding signal peptide) was used for A/H3N2 viruses sequence analysis [22].

### 2.11 Antigenic Characteristics of HA Segments Based on Amino Acid Substitution

The five HA antigenic sites (Sa, Sb, Ca1, Ca2, and Cb) of influenza A/H1N1pdm09 [23] and the five antigenic epitopes (Epitopes A, B, C, D, and E) of influenza A/H3N2 [24] as well as the NA antigenic sites [25] were examined.

### 2.12 Prediction of Potential Glycosylation Sites

Influenza viruses alter the potential N linked glycosylation (PNG) sites or positions in surface glycoproteins to escape host immune response and hence facilitate continued replication [26]. PNG sites were predicted using NetNGlyc 1.0. [27] from the Technical University of Denmark (DTU) (https://services.healthtech.dtu.dk/service.php? NetNGlyc‐1.0; accessed on 02 Feb 2023). The PNG sequon used to predict sites is Asparagine‐X‐Serine/Threonine‐X (NX[T/S]X), where X can be any amino acid except Aspartic acid or Proline. Threshold values above 0.5 indicate glycosylation.

### 2.13 Statistical Analysis

Statistix version 10 used for statistical analysis and graphics representations was generated by using Microsoft Excel (Office 365).

## 3 Results

### 3.1 Study Population

A total of 6316 patients tested as part of ILI‐SARI surveillance from January 2017 to December 2019 were included of which 1428 (22.6%) tested positive for influenza A/B viruses. Males represented 53.2% (759/1428) and females 46.8% (669/1428). Participants had a median age of 29 years (range 0 to 97 years).

### 3.2 Seasonal Distribution of Influenza A Viruses

Influenza A PCR positives were observed for 92.6% (1322/1428) of the study population. Overall, influenza A/H1N1pdm09 strains prevailed at 62.5% (826/1322) while A/H3N2 strains represented only 37.1% (491/1322) (Table 1). In 2017, A/H3N2 dominated (259/463; 55.9%) followed by A/H1N1pdm09 at 43.6% (202/463). Although influenza A viruses dominated at 66.4% (71/107) in 2018; overall influenza viruses circulated at a lower prevalence (11.2%; 107/955) compared to 2017 and 2019. In 2018, A/H1N1pdm09 was detected at 53.5% (38/71) and A/H3N2 at 42.3% (30/71). In 2019 influenza A/H1N1pdm09 viruses dominated (586/788; 74.4%) followed by A/H3N2 (202/788; 25.6%).

**TABLE 1:** Respiratory samples processed as part of Influenza surveillance during the period 2017–2019 with corresponding A/H1N1pdm09 and A/H3N2 PCR positive cases detected.

Year	Respiratory samples processed	Influenza A/B positives *n* (%)	Influenza A positives *n* (%)	H1N1pdm09 *n* (%)	H3N2 *n* (%)	A not typed. *n* (%)
2017	1523	496 (32.6)	463 (93.3)	202 (43.6)	259 (55.9)	2 (0.4)
2018	955	107 (11.2)	71 (66.4)	38 (53.5)	30 (42.3)	3 (4.2)
2019	3838	825 (21.5)	788 (95.5)	586 (74.4)	202 (25.6)	0
**Total**	**6316**	**1428 (22.6)**	**1322 (92.6)**	**826 (62.5)**	**491 (37.1)**	**5 (0.4)**

### 3.3 Influenza a Virus Cultures

Virus isolation was attempted on approximately 90.0% (1159/1322) of influenza positive samples. However, virus isolation was successful for only 11.0% (128/1159) of which 78.1% (100/128) was A/H1N1pdm09 and 21.9% (28/128) A/H3N2. The average influenza A M gene PCR Ct value was lower at 24.6, 25.1, and 24.2, respectively in 2017, 2018, and 2019, among samples which generated virus isolates compared to those that were culture negative (1031/1159; 89.1%). Maximum Ct values for samples that were culture negative ranged from 36 to 38 (28/1031; 2.7%), but some low values of 16 to 20 (55/1033; 5.3%) were also observed.

### 3.4 Influenza A Viruses' Genome Amplification and Sequencing

Influenza A virus genome amplification and sequencing were done for 118/126 (93.7%) virus isolates of which A/H1N1pdm09 represented 77.1% (91/118) and A/H3N2 22.9% (27/118). Quality control of sequence reads with FastQC for all samples showed that average Phred scores of 20 were obtained. Genome coverage ≥ 80% was obtained for 49/91 (53.8%) A/H1N1pdm09 isolates (Table S2a). For influenza A/H3N2 isolates, 21/27 (77.8%) had ≥ 80% genome coverage (Table S2b).

### 3.5 Phylogenetic Analysis of Influenza A/H1N1pdm09 Viruses HA and NA Gene Segments

**FIGURE 1:**
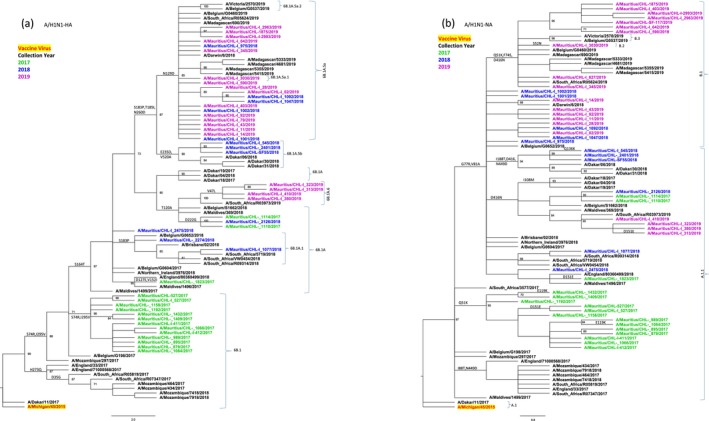
Maximum‐likelihood (a) HA and (b) NA genes phylogenetic trees from influenza A/H1N1pdm09 viruses detected in Mauritius, 2017–2019. Statistical confidence in branch placements were determined using 1000 bootstrap replicates and significant clusters were defined by bootstrap values > 70%. The vaccine strain is colored bright red. Mauritius strains from each year are indicated by different colors as shown in the color key. The key amino acid substitutions defining clades and subclades are indicated on the branches.

The HA phylogenetic tree (Figure 1a) together with Nextclade analysis revealed that A/H1N1pdm09 virus HA genes from the 2017–2019 clusters in 6B.1 genetic lineage subgroups. A/H1N1pdm09 6B.1 subclades with varying annual dominance were as follows: 6B.1 (2017: 13/16, 81.3%), 6B.1A.5a (2018: 5/12, 41.7%), and 6B.1A.5a (2019: 14/20, 70.0%).

The influenza A/H1N1pdm09 virus NA genes (48/49) from 2017 to 2019 belong to three genetic subgroups (Figure 1b): A.1.1 (27/48, 56.3%) circulated 2017–2019, while B.1 (20/48, 41.7%) and B.2 (1/48,2.1%) circulated in 2018 and 2019. Clade A.1.1 dominated in 2017 (16/16, 100%) while in 2018, clade A.1.1 was observed at 54.5% (6/11) and B.1 at 45.5% (5/11). In 2019, clade B.1 dominated (15/20, 75%). The A/Mauritius/CHL‐I 79/2019 isolate had a premature stop codon in NA and were excluded.

### 3.6 Amino Acid Substitutions Among Influenza A/H1N1pdm09 viruses' HA, NA, PA, and M Proteins and Predicted Resistance to Antivirals

Predicted amino acids of influenza A/H1N1pdm09 viruses' HA, NA, M1, M2, and PA proteins were compared with the reference strain A/Michigan/45/2015 (Figure S1). Alignment of HA amino acid sequences of influenza A/H1N1pdm09 viruses from 2017 to 2019 showed a total of 36 amino acid substitutions in HA1 (2017: 13, 2018: 21, and 2019: 17) and a total of 11 amino acid substitutions in HA2 (2017: 4, 2018: 7 and 2019: 6) (Figure S1A) compared with A/Michigan/45/2015. In 2017, A/H1N1pdm09 HA1 amino acid substitutions S74R and I295V characteristic of the 6B.1 subgroup were observed. The 6B.1A subcluster that emerged had additional amino acid substitutions T120A, S164T, D222G in HA1, and D112N in HA2 (HA2 numbering).

In 2018, subgroup 6B.1 completely phased out with continued circulation of 6B.1A and emergence of 6B.1A.1, 6B.1A.5a, and 6B.1A.5b subgroups. Subgroup 6B.1A.5a had amino acid substitutions N129D, S183P, T185I, and N260D as the dominant lineage while the 6B.1A.5b subgroup also had substitutions E235D in HA1 and V193A in HA2. The D222G substitution were observed in 6B.1A lineage A/H1N1pdm09 viruses from three patients (two female and one male) that were hospitalized with SARI of which one was in critical care (male). In 2019, A/H1N1pdm09 HA subgroup 6B.1A.5a also predominated with occurrence of a few 6B.1A.6 strains, which was characterized by an additional amino acid substitution, V47L.

A total of 34 amino acid substitutions (2017: 18, 2018: 29 and 2019: 24) were observed in A/H1N1pdm09 NA amino acid sequences (Figure S1B) from 2017 to 2019 when compared to A/Michigan/45/2015. In 2017, subclade A.1.1 defined by the G77R, I88T and N449D substitutions prevailed exclusively (16/16, 100%), whereas in 2018 an equal distribution of clade A.1.1 (6/11, 54.5%) and clade B.1 (5/11, 45.5%) were observed. In 2019, clade B.1 (15/20, 75%) defined by Q51K, F74S, V81A, and D416N amino acid substitutions dominated. In 2019, A/Mauritius/CHL‐I_3030/2019, belonged to the B.2 sublineage characterized by the S52N substitution. Two significant substitutions Q136K (2018: 1/47) and D151E (2017 and 2019: 4/47) were observed for subclade A.1.1.

Amino acid substitution I80V was predominant in M1 (9/48) (Figure S1C), while M2 amino acid substitutions (Figure S1D) in 13/48 viruses were as follows: R12K (3/48) and I28T (3/48) in 2017–2018; and Q81H (5/48), E70D (1/48), and G21V (1/48) among 2019 viruses. Lastly, amino acids substitutions in PA (Figure S1E) for 11/18 A/H1N1pdm09 viruses were: P68S (2/11); F105L (1/11); M311V (2/11); K353R (1/11); V407I (1/11); N614H (2/11); V669G (3/11); and I690V (1/11).

### 3.7 Characterization of Antigenic Regions

In A/H1N1pdm09 virus HA antigenic sites (Table 2), the following substitutions were observed: N129D (20/47; 42.6%), N162S resulting in the loss of PNG site (1/47; 2.1%), and S164T, which maintained the PNG (34/47; 72.3%) in Sa. In antigenic site Sb D187N (1/47; 2.1%) and S190R (2/47; 4.3%), substitutions were observed with E224K/G (3/47; 4.1%) in Ca2 and S91R (47/47:100%) in Cb.

**TABLE 2:** Amino acid substitutions in the HA1 region among influenza A/H1N1pdm09 viruses from Mauritius, 2017–2019, compared to vaccine virus A/Michigan/45/2015. A dot (.) represents a conserved amino acid.

A(H1N1)pdm09	HA1							
Antigenic sites	Cb	Sa			Sb			Ca2
**Amino acid position (H1 numbering)‐17aa**	**74**	**129**	**162**	**164**	**187**	**189**	**190**	**224**
**A/Michigan/45/2015**	**S**	**N**	**N**	**S**	**D**	**Q**	**S**	**E**
**6B.1**	**R**	**.**	**S**	**.**	**.**	**.**	**.**	**.**
**6B.1A**	**R**	**.**	**.**	**T**	**.**	**.**	**.**	**.**
**6B.1A.1**	**R**	**.**	**.**	**T**	**.**	**.**	**.**	**.**
**6B.1A.5a**	**R**	**D**	**.**	**T**	**N**	**.**	**R**	**K/G**
**6B.1A.5a.1**	**R**	**D**	**.**	**T**	**A**	**E**	**.**	**.**
**6B.1A.5b**	**R**	**.**	**.**	**T**	**.**	**.**	**.**	**K**
**6B.1A.6**	**R**	**.**	**.**	**T**	**.**	**.**	**.**	**.**

### 3.8 Phylogenetic Analysis of Influenza A/H3N2 Viruses HA and NA Gene Segments

**FIGURE 2:**
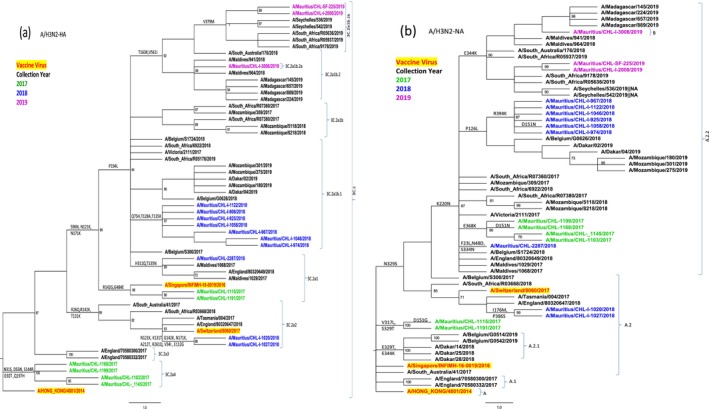
Maximum‐likelihood phylogenetic trees for (a) HA and (b) NA gene segments from influenza A/H3N2 viruses detected in Mauritius during 2017–2019. Statistical confidence in branch placements were determined using 1000 bootstrap replicates and significant clusters were defined by bootstrap values > 70%. Mauritius strains from each year are indicated by different colors as shown in the color key and relevant vaccine strains are colored bright red. Key amino acid substitutions defining clades and subclades are indicated on branches.

Phylogenetic analysis of the Mauritius A/H3N2 HA gene segments revealed that 70.4% (21/27) of isolates belonged to 3C.2a clade with varying annual dominance (Figure 2a): 3C.2a4 (2017: 5/7, 71.4%), 3C.2a1b.1 (2018: 8/11, 72.7%) and 3C.2a1b.2b (2019: 2/3, 66.7%). The 2017 predominant 3C.2a4 subclade clustered closely with the vaccine virus A/Hong Kong/4801/2014, while the 2017 3C.2a1 clade clustered closely with the 2018 vaccine strain, A/Singapore/INFIMH‐16‐0019/2016. In 2018, the prevalent clade 3C.2a1b.1 evolved away from the vaccine strain and the prevalent 2019 3C.2a1b.2 sublineages were also remotely located from the 2019 A/Switzerland/8060/2017 vaccine strain. Two Mauritius isolates sampled in November 2018; A/Mauritius/CHL‐I‐1020/2018 and A/Mauritius/CHL‐I‐1027/2018 of the 3C.2a2 clade clustered closely to the A/Switzerland/8060/2017 vaccine strain.

N2 gene sequences from 2017 to 2019 cluster in two genetic subgroups A.2 and A.2.2 (Figure 2b). Clade A.2.2 dominated (2017: 5/7,71.4%), (2018: 10/11, 90.9%), (2019: 2/3, 66.7%). In 2019, genetic subgroup B also circulated (1/3, 33.3%).

### 3.9 Amino Acid Substitutions Across Influenza A/H3N2 Viruses' HA, NA, PA, and M Proteins and Their Predicted Resistance to Antivirals

In 2017, the co‐circulating 3C.2a1 and 3C.2a4 shared common amino acid mutation S96N, R142G, while the 3C.2a4 acquired additional substitutions N31S, D53N, S144R, I192T, and Q197H. The 3C.2a2 lineage in 2018 was defined by a series of amino acid substitutions in HA: N121K, K131T, G142K, N171K, A212T, R261Q (HA1) and V34I and E112G (HA2) and the dominant 3C.2a1b.1 lineage (2018: 8/11, 72.7%) was characterized by amino acid substitutions E62G, Q75H, K92R, T128A, T135N, and H311Q (HA1) and E107G (HA2). The 194P and 160K (Figure S2A) substitutions in epitope B occurred during the egg‐propagation of influenza A/H3N2 vaccine strains and were excluded in analysis [28]. Out of the 48 amino acid substitutions in A/H3N2 HA segments, 22 amino acid substitutions were observed in A/H3N2 antigenic sites: four in site A; three in site B; one in site C; six in site D and four in site E (Table 3). In 2017, the dominant 3C.2a4 virus strain differed from the A/Hong Kong/4801/2014 vaccine strain by 10 amino acid substitutions across all the five antigenic sites: site A (K131T, R142G, S144R), site B (N158K and I192T), site C (D53N), site D (S96N, N171K, L177M), and site E(Q261R). In 2018, the clade 3C.2a1b.1 was most prevalent, and it differed from the A/SingaporeINFIMH‐16‐0019/2016 vaccine strain by six amino acid substitutions across four antigenic sites: site A (T135N), site B (T128A), site D (I214T), and site E (E62G, Q75H, K92R). In 2019, the 3C.2a1b.2b clade differed from the A/Switzerland/8060/2017 vaccine strain by seven amino acid substitutions across three antigenic sites: site A (K142G), site D (S96N, N121K, N171K), and site E (E62G, K92R, Q261R) (Table 3).

**TABLE 3:** Amino acid substitutions in the HA1 region among influenza A/H3N2 viruses from Mauritius, 2017–2019, compared to the relevant vaccine strains for each year. A dot (.) represents a conserved amino acid.

A(H3N2)	HA1																					
Antigenic sites	C	E	E	E	E	E	D	D	B	A	A	A	A	B	B	D	D	B	B	D	D	E
FluSurver numbering/H3 numbering (With Sig Peptide) + 16aa	69	78	91	99	108	110	112	137	144	147	151	158	160	174	176	187	193	208	210	228	230	277
Amino acid position (H3 numbering)‐16aa	53	62	75	83	92	94	96	121	128	131	135	142	144	158	160	171	177	192	194	212	214	261
A/Hong Kong/4801/2014	D	E	Q	K	K	Y	S	N	T	K	T	R	S	N	K	N	L	I	P	A	I	Q
3C.2a1	**.**	**.**	**.**	**.**	**.**	**.**	N	K	**.**	**.**	N	G	**.**	K	T	K	M	**.**	L	**.**	**.**	**.**
3C.2a4	N	**.**	**.**	**.**	**.**	**.**	N	**.**	**.**	T	**.**	G	R	K	T	K	M	T	L	**.**	**.**	R
A/SingaporeINFIMH‐16‐0019//2016	D	E	Q	K	K	Y	N	N	T	K	T	G	S	N	K	K	L	I	P	A	I	R
3C.2a1	**.**	**.**	**.**	**.**	R	**.**	**.**	**.**	**.**	**.**	**.**	R	**.**	**.**	**.**	**.**	**.**	**.**	L	**.**	**.**	**.**
3C.2a2	**.**	**.**	**.**	**.**	**.**	**.**	**.**	K	A	T	**.**	K	**.**	**.**	**.**	N	**.**	**.**	L	T	**.**	Q
3C.2a1b.1	**.**	G	H	**.**	R	**.**	**.**	**.**	A	**.**	N	**.**	**.**	**.**	R/T	**.**	**.**	**.**	L	**.**	T	**.**
A/Switzerland/8060/2017	D	E	Q	K	K	Y	S	N	T	K	T	K	S	N	K	N	L	I	P	A	I	Q
3C.2a1b.2a	**.**	G	**.**	E	R	N	N	K	**.**	**.**	**.**	G	**.**	**.**	**.**	K	**.**	**.**	L	**.**	**.**	R
3C.2a1b.2b	**.**	G	**.**	**.**	R	**.**	N	K	**.**	**.**	**.**	G	**.**	**.**	T	K	**.**	**.**	L	**.**	**.**	R

*Note:* P194L and K160T being scored as differences from the egg‐adaption seen in the vaccine viruses.

The NA sequence analysis revealed 41 amino acid substitutions among the 21 Mauritian A/H3N2 viruses. The most common were P126L, I212V, K220N, V303I, and N329S (Figure S2B). Significant substitutions that may affect NA functionality were D151G (1/21) and D151N (3/21), S245N (6/21), S247T (6/21), N329T (2/21), N329S (15/21), and E368K (5/21). No substitutions were observed in the A/H3N2 M1 gene segments while the M2 gene segments displayed five amino acid substitutions (Figure S2C). The most common M2 substitution was A21V and the significant mutation V27A were also observed but the amino substitutions L26F, A30T, A30V, S31N, G34E, and L38F responsible for adamantane resistance [29] was not observed. One of the three subunits encoding the RNA‐dependent RNA polymerase PA had amino acid substitutions in 8/21 sequences namely, S60T, N272S, G101E, L105F, V668I, K158R, V565M, and L586I (Figure S2D).

### 3.10 Predicted N‐Linked Glycosylation Sites in A/H1N1pdm09 and A/H3N2 Viruses

The number of N‐linked glycosylation sites (PNGs) in HA of A/H1N1pdm09 viruses from 2017 to 2019 remained mostly at 7 (44/48). PNGs in HA1 were the following: N^10^NS, N^23^VT, N^87^GT, N^162^Q(S/T), N^276^NTT, N^287^TS; and N^154^GT were observed in HA2. The number of PNGs across NA protein sequences of A/H1N1pdm09 (46/48) remained constant at 8. These were at positions N^42^QS, N^50^QS, N^58^NT, N^63^QT, N^68^IS, N^88^SS, N^146^GT, and N^235^GS.

The PNGs in the HA of A/H3N2 viruses were mostly 12 (9/21) with a range of 10 to13. The dominant A/H3N2 clade 3C.2a4 (5/7; 80.0%) detected in 2017 had 11 PNGs in the HA1 domain (N^8^ST, N^22^GT, N^38^AT, N^45^SS, N^63^CT, N^122^ES, N^126^WT, N^133^GT, N^165^VT, N^246^ST, and N^285^GS) and N^154^GT in the HA2 domain. Thirteen of the A/H3N2 (61.9%, 13/21) viruses had lost the PNG at position 158 (N^158^YT) of HA1 due to substitutions (Table S3). Three strains from 2017 had a N158K substitution whereas two strains from 2017, 5 from 2018, and 2 from 2019 had T160K substitutions that resulted in loss of this PNG. In addition, another strain from 2018 had a T160R substitution as cause for the PNG loss. The dominant clade in 2018, 3C.2a1b.1 (8/11) displayed 11 (4/8); N^8^ST, N^22^GT, N^38^AT, N^45^SS, N^63^CT, N^122^ES, N^158^YT, N^165^VT, N^246^ST, N^285^GS, N^154^ET) and 10 (3/8) PNGs with the loss of N^158^YT in the latter group. In 2019, subclades 3C.2a1b.2b (2/3) and 3C.2a1b.2a (1/3) had 12 and 13 PNG sites, respectively. The number of PNGs among the NA sequences of A/H3N2 was 8 for 16/21 (76.2%) viruses at the following positions: N^61^IT, N^70^TT, N^86^WS, N^146^NT, N^200^AT, N^234^GT, N^245^AT, and N^367^KT. Substitutions at NA position 329 among 17/21 (81.0%) A/H3N2 viruses that resulted in loss of the PNG site were N329S (15/17; 88.2%) and N329T (2/17; 11.8%).

## 4 Discussion

This study investigated the circulation of influenza A/H1N1pdm09 and A/H3N2 viruses in Mauritius between 2017 and 2019 with an overall detection rate for influenza A/B viruses of 22.6% (1428/6316) of which the majority (1322/1428; 92.6%) was influenza A. Influenza A/H1N1pdm09 dominated with an overall prevalence of 62.5% (826/1322) and A/H3N2 for 37.1% (491/1322); however, A/H3N2 dominated in 2017 (259/463;55.9%), and sporadic influenza virus activity was observed in Mauritius in 2018. Severe influenza seasons with A/H3N2 viruses were reported for 2017 and 2019 in South Africa [30] and Australia [31].

Phylogenetic analysis revealed that clade 6B dominated in Mauritius during 2017–2019 like Europe, Asia, and Africa where clade 6 was also prevalent during this period [32, 33]. All 47 A/H1N1pdm09 viruses HAs exhibited the S74R, S164T, and I295V amino acid substitutions characteristic of the 6B clade. These substitutions are thought to alter the salt bridge pattern and stability of the membrane fusion process [34]. The A/H1N1pdm09 6B.1 lineage viruses have shared S74R and I295V substitutions, which were also observed in strains from India, Bulgaria, and Kenya [32, 35, 36, 37]. One sample A/Mauritius/CHL_879/2017 displayed N162S substitution causing a loss of the glycosylation site [38]. Despite low influenza A/H1N1 activity in 2018, the emerged clade 6B.1A.5a had the most amino acid substitutions (S74R, N129D, S164T, D187N, S190R, E224K/G) in four antigenic sites and persisted in 2019 in Mauritius as well as in Europe [39]. Notably, substitution D222G (3/47) in 6B.1A subgroup viruses from patients that were admitted with SARI in 2017 and 2018. The D222G mutation has been linked to an increase in virulence of the A/H1N1pdm09 strain during the 2009 pandemic [40].

The observation that the 6B.1 clade in 2017 clustered near the vaccine strain A/Michigan/42/2015 while the 2018–2019 subclades (6B.1A, 6B.1A.1, 6B.1A.5a, 6B.1A.5a.1, 6B.1A.5b, 6B.1A.6) where further away which may suggest that similar to in Europe during 2017–2018 low vaccine effectiveness would have been expected [41]. However, in this period it was reported that A/H1N1pdm09 viruses in Mauritius remained antigenically like A/Michigan/45/2015 based on post‐infection ferret antisera neutralization titres [42].

A/H1N1pdm09 NA proteins for circulating clades A.1.1, B.1, and B.2 were characterized by G77R, I88T, and N449D substitutions. Other notable amino acid substitutions included E119K (2/47 in 2017), Q136K (1/47 in 2018), and D151E (4/47 in 2017 and 2019). E119K and D151E substitutions were previously linked to oseltamivir drug resistance [43, 44]. Q136K was associated with reduced binding affinity to neuraminidase inhibitors [45] but has not been reported recently [30]. Recently, Leung et al. [46] reported detection of the I223V and S247N substitutions associated with reduced susceptibility to neuraminidase inhibitors in A/H1N1pdm09 viruses from Hong Kong and observed emergence of these amino acid substitutions from 2023 in global data [47]. However, the frequency of oseltamivir resistance associated with the H275Y substitution was still low during the same period whereas single and combined I223V and S247N substitutions were more prevalent especially in Bangladesh [47]. These substitutions were not present in the NAs of A/H1N1pdm09 viruses identified in Mauritius in 2017–2019.

During 2017–2019, influenza A/H3N2 viruses from Mauritius displayed greater genetic heterogeneity. Genetic clade 3C.2a subdivided into six subclades with co‐circulation of two or more subclades during the same year. In 2017, subclade 3C.2a4 and 3C.2a1 co‐circulated with 3C.2a4 clustering close to A/Hong Kong/4801/2014, while 3C.2a1 clustered near the 2018 A/Singapore/INFIMH‐16‐0019/2016 vaccine virus. In 2018, the subclade 3C.2a1b.1 emerged, and its subclades continued to circulate in 2019. The predominance of 3C.2a1b clade from 2018–2019 was also noted in Europe [39] and Thailand [34].

The 3C.2a1 strain shared substitutions S96N, N121K, R142G, N171K in HA1 and G484E (155/HA2 numbering) in HA2 segment with strains from other countries [48, 49, 50]. The 3C.2a4 sublineage had five additional substitutions: N31S, D53N, S144R, I192T, and Q197H. Notably, S144R may alter antigenic epitopes at receptor sites contributing to immune escape [50, 51] and viruses with the I192T substitution showed reduced neutralization by A/Hong Kong/4801/2014 (H3N2) vaccine induced antibodies [52]. The 3C.2a1b.1 variant, which dominated in 2018 (72.7%, 8/11) in Mauritius, had six amino acid substitutions across four antigenic sites A, B, D, and E including the presence of T135K known to result in the loss of a PNG site in antigenic site A [52]. This substitution contributed to the drift from the A/SingaporeINFIMH‐16‐0019/2016 vaccine strain. Similarly, the loss in PNG site caused by the T160K substitution in antigenic site B of egg‐adapted A/H3N2 vaccine strains changes the neutralizing antibody profile it induces [53] and has been associated with reduced vaccine effectiveness [54]. Vaccine effectiveness against A/H3N2 viruses was only 28% during the 2017–2018 season in Europe where the 3C.2a1 lineage viruses circulated [41]. The severe H3N2 seasons reported in Australia and other Southern Hemisphere countries may also have been the result of decreased vaccine effectiveness against 3C.2a1 lineage viruses for which poor neutralization by ferret antibodies against A/Switzerland/8060/2017 vaccine strain have been reported [55]. Significant substitutions in A/H3N2 NA sequence analysis that may affect NA functionality were D151G (1/21) and D151N (2/21). Polymorphism at NA positions 151 is likely an artefact of MDCK cell culture in 3C.2a viruses, as observed in isolates from the United Kingdom [56].

The loss of the PNG site at position 158 (11/21; 52.4%) in antigenic site B of A/H3N2 viruses from Mauritius was unexpected as all influenza virus isolates were generated in MDCK cells. A/H3N2, 3C.3a subclades have demonstrated greater amino acid substitutions on passage in conventional MDCK cells than in MDCK‐ SIAT1 cells which altered their antigenic and receptor binding properties [56]. For most A/H3N2 viruses from this study (9/13; 69.2%), the PNG loss was the result of T160K substitution, which has been characterized as an adaptive mutation for egg‐propagated influenza viruses (Table S3). Notably, the change is from an uncharged to positively charged residues [57]. Structural modelling focused on positions 158 to 160 of the HA1 region of A/H3N2 viruses highlighted that these substitutions result in change of the HA structure, which will impact receptor binding and antigenicity [57]. A/H3N2 viruses with the N158 genotype, which confers a glycosylation site were first identified in early 2010. Our observation of three A/H3N2 viruses from 2017 with the K158 genotype is unusual and indicates reversion to a genotype that dominated in 2000 to 2008 [57]. The substitution at position 158 has also been linked to reduced ability of monoclonal antibodies to block receptor binding [53] and exhibition of higher viral pathogenicity [58]. Understanding the gain and loss of N‐linked glycosylation site is likely to impact vaccine strain selection or design strategies.

In this study, we observed loss of the 329 PNG site in the NA of the majority (81.0%, 17/21) of A/H3N2 viruses. Similarly, Ge et al. [59] reported an increase in A/H3N2 viruses without the 329 PNG site in NA from 2015 onwards with all having lost the PNG by 2018 to 2020 with resultant lower cross protection by antibodies [59]. They proposed that the A/H3N2 viruses' NA be considered in future vaccine strain selection and design strategies. NA genotypes N329K has been associated with reduced susceptibility to oseltamivir and zanamivir [43] although Hussain et al. [60] examined the 329 site and showed no change in sialidase activity for viruses involving N329S/T substitutions observed in this study.

## 5 Strengths and Limitations

This study represents the first report on the molecular epidemiology of influenza A viruses in Mauritius, focusing on the 2017–2019 period. However, there are some limitations. Firstly, influenza A viruses were propagated from only 11% of the samples. The lack of growth by culture for specimens with early Ct values demonstrates the caveat of ascribing a proportionality between the Ct value and viable material in the specimen. Diverse factors affect Ct value irrespectively and independently of the amount of cultivable and viable influenza virus material presents in respiratory swabs while other external factors such as cold chain for sample handling and storage may compromise the successful viral culture. Secondly, while Oxford Nanopore Technologies' long‐read sequencing technology has significantly advanced genomics, data is still challenged by high error rates due to insertions and deletions. In this study the R9.4.1 flow cell was used whereas the newer R 10.4 flow cells were reported to improve accuracy [61].

Another limitation was the inability to sequence the original clinical specimens and therefore we could not verify whether the Q136K and D151E amino substitutions, for example, were genuine or arose during virus culture. Despite these limitations, the findings offer a crucial first insight into the genetic evolution of influenza A viruses in Mauritius during the 2017–2019 pre‐COVID‐19 pandemic period.

## 6 Conclusions

In summary, our study provides a comprehensive overview of the genetic diversity, amino acid substitutions, and evolutionary pattern of circulating influenza A/H1N1pdm09 and A/H3N2 in Mauritius from 2017 to 2019. We found no clear annual pattern of influenza activity in Mauritius and identified numerous amino acid substitutions among the strains highlighting the continued genetic drift in circulating influenza A viruses during 2017 to 2019. Notably, substitutions affecting sensitivity to neuraminidase inhibitors were limited in both influenza A/H1N1pdm09 and A/H3N2 viruses whereas substitutions that resulted in loss of PNGs in A/H3N2 viruses' HA (positions 158 and 160) and NA (position 329) could impact antigenicity and sensitivity to neutralizing antibodies for these viruses. This underscores the need for routine influenza surveillance and timely genomic monitoring where this study outcomes serve as baseline for future research on influenza viruses in Mauritius. Integrating genetic and epidemiological data will facilitate informed decisions regarding optimal influenza vaccine composition, antiviral treatment strategies and the timing of influenza vaccinations.

## Author Contributions

**Magalutcheemee Ramuth:** conceptualization, investigation, writing – original draft, methodology, writing – review and editing, formal analysis, visualization, data curation, resources, software. **Janaki Sonoo:** supervision, writing – review and editing, resources. **Fhooblall Mokshanand:** supervision, writing – review and editing. **Belinda Herring:** writing – review and editing, resources. **Tessema Sofonias:** writing – review and editing, resources. **John McCauley:** validation, writing – review and editing, resources, methodology, funding acquisition. **Ashwamed Dinasing:** writing – review and editing, resources. **Florette K. Treurnicht:** conceptualization, supervision, funding acquisition, writing – review and editing, project administration, resources, methodology, validation, visualization, data curation, software.

## Conflicts of Interest

The authors declare no conflicts of interest.

### Peer Review

The peer review history for this article is available at https://www.webofscience.com/api/gateway/wos/peer‐review/10.1111/irv.70108.

## Supporting information


**Table S1.** Influenza virus vaccine strains used in 2017–2019 influenza seasons in the Southern and Northern Hemispheres (summarized from https://www.who.int/teams/global‐influenza‐programme/vaccines/who‐recommendations). Vaccine components that were changes from the previous season are underlined.

**Figure S1.** Mutational analysis of haemagglutinin (HA), neuraminidase (NA), matrix (M), and polymerase (PA) protein of A/H1N1pdm09 (*N* = 48) using FluSurver and using the Southern Hemisphere vaccine strain A/Michigan/45/2015 as reference. Red colored substitutions are known to alter the virulence of the virus and cause strong drug resistance. Orange colored substitutions occur at sites known to be involved in drug binding or alter host‐cell specificity.

**Figure S2.** Mutational analysis of haemagglutinin (HA), neuraminidase (NA), matrix (M), and polymerase (PA) proteins of A/H3N2 (*N* = 21) using FluSurver and compared to the reference sequence for 2017–2019 flu seasons; A/Hong Kong/4801/2014, A/Singapore/INFIMH‐16‐0019/2016 and A/Switzerland/8060/2017. Red colored substitutions are known to alter the virulence of the virus and cause strong drug resistance. Orange colored substitutions occur at sites known to be involved in drug binding or alter host‐cell specificity.

**Table S2.** GISAID ID and acknowledgement for reference sequences used in study (Excel).

**Table S3.** N‐linked glycosylation sites in the haemagglutinins and neuraminidases of influenza A/H1N1pdm09 and A/H3N2 viruses from Mauritius, 2017–2019 (Excel).

## Data Availability

Influenza A viruses' genome sequences from this study and associated metadata in this dataset are published in GISAID's EpiFlu database. GISAID IDs are provided in Supplemental Table S2.
