# Supplementary material for: Molecular Evolution of Influenza A Viruses From Mauritius, 2017–2019
Source: Influenza Other Respir Viruses. 2025 May 13;19(5):e70108. doi: 10.1111/irv.70108 (PMC12074737; doi:10.1111/irv.70108)
Supplement: Supplementary file 2 — Figure S1. Mutational analysis of haemagglutinin (HA), neuraminidase (NA), matrix (M), and polymerase (PA) protein of A/H1N1pdm09 (N = 48) using FluSurver and using the Southern Hemisphere vaccine strain A/Michigan/45/2015 as reference. Red colored substitutions are known to alter the virulence of the virus and cause strong drug resistance. Orange colored substitutions occur at sites known to be involved in drug binding or alter host‐cell specificity. [file IRV-19-e70108-s005.pdf]

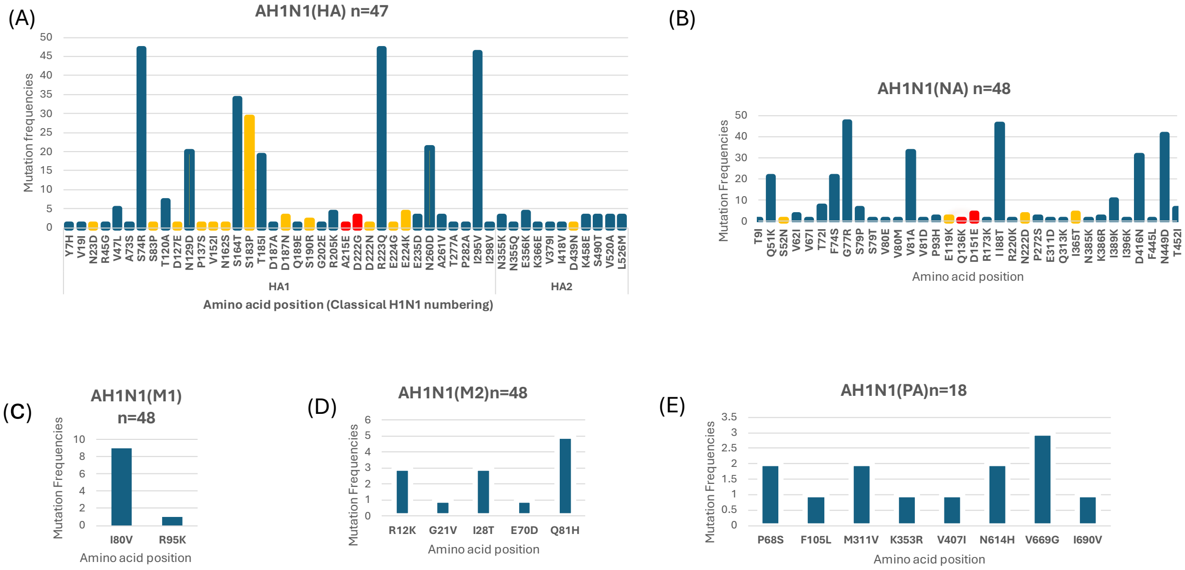

Supplemental Figure S1. Mutational analysis of haemagglutinin (HA), neuraminidase (NA), matrix (M) and polymerase (PA) protein of A/H1N1pdm09 (N=48) using FluServer and using the Southern Hemisphere vaccine strain A/Michigan/45/2015 as reference. Red coloured substitutions are known to alter the virulence of the virus and cause strong drug resistance. Orange coloured substitutions occur at sites known to be involved in drug binding or alter host-cell specificity.
