# Supplementary material for: Molecular Evolution of Influenza A Viruses From Mauritius, 2017–2019
Source: Influenza Other Respir Viruses. 2025 May 13;19(5):e70108. doi: 10.1111/irv.70108 (PMC12074737; doi:10.1111/irv.70108)
Supplement: Supplementary file 4 — Table S2. GISAID ID and acknowledgement for reference sequences used in study (Excel). [file IRV-19-e70108-s002.docx]

**Supplementary tables and figures**

**Supplemental Table S1.** Influenza virus vaccine strains used in 2017-2019 influenza seasons in the Southern and Northern Hemispheres (summarised from https://www.who.int/teams/global-influenza-programme/vaccines/who-recommendations). Vaccine components that were changes from the previous season are underlined.

| **Southern Hemisphere** | | |
| --- | --- | --- |
| **Year** | **Trivalent vaccine** | **Quadrivalent vaccine** |
| 2017 | A/Michigan/45/2015 (H1N1pdm09)-like virus  A/Hong Kong/4801/2014 (H3N2)-like virus  B/Brisbane/60/2008-like virus (*B/Victoria-like) | A/Michigan/45/2015 (H1N1pdm09)-like virus  A/Hong Kong/4801/2014 (H3N2)-like virus  B/Brisbane/60/2008-like virus (*B/Victoria-like)  B/Phuket/3073/2013-like virus (**B/Yamagata-like) |
| 2018 | A/Michigan/45/2015 (H1N1pdm09)-like virus  A/Singapore/INFIMH-16-0019/2016 (H3N2)-like virus  B/Phuket/3073/2013-like virus | A/Michigan/45/2015 (H1N1pdm09)-like virus  A/Singapore/INFIMH-16-0019/2016 (H3N2)-like virus  B/Phuket/3073/2013-like virus  B/Brisbane/60/2008-like virus |
| 2019 | A/Michigan/45/2015 (H1N1pdm09)-like virus  A/Switzerland/8060/2017 (H3N2)-like virus  B/Colorado/06/2017-like virus (*B/Victoria-like) | A/Michigan/45/2015 (H1N1pdm09)-like virus  A/Switzerland/8060/2017 (H3N2)-like virus  B/Colorado/06/2017-like virus (*B/Victoria-like)  B/Phuket/3073/2013-like virus. |
| **Northern Hemisphere** | | |
| **Year** | **Trivalent vaccine** | **Quadrivalent vaccine** |
| 2017 | A/California/7/2009 (H1N1pdm09)-like virus  A/Hong Kong/4801/2014 (H3N2)-like virus  B/Brisbane/60/2008-like virus | A/California/7/2009 (H1N1pdm09)-like virus  A/Hong Kong/4801/2014 (H3N2)-like virus  B/Brisbane/60/2008-like virus  B/Phuket/3073/2013-like virus |
| 2018 | A/Michigan/45/2015 (H1N1pdm09)-like virus  A/Hong Kong/4801/2014 (H3N2)-like virus  B/Brisbane/60/2008-like virus | A/Michigan/45/2015 (H1N1pdm09)-like virus  A/Hong Kong/4801/2014 (H3N2)-like virus  B/Brisbane/60/2008-like virus  B/Phuket/3073/2013-like virus |
| 2019 | A/Michigan/45/2015 (H1N1pdm09)-like virus  A/Singapore/INFIMH-16-0019/2016 (H3N2)-like virus  B/Colorado/06/2017-like virus (*B/Victoria-like) | A/Michigan/45/2015 (H1N1pdm09)-like virus  A/Singapore/INFIMH-16-0019/2016 (H3N2)-like virus  B/Colorado/06/2017-like virus (B/Victoria-like)  B/Phuket/3073/2013-like virus |

*B/Victoria/2/87 lineage, **B/Yamagata/16/88 lineage

**Supplemental Table S2.** GISAID ID and acknowledgement for reference sequences used in study (Excel)

**Supplemental Table S3.** N-linked glycosylation sites in the haemagglutinins and neuraminidases of influenza A/H1N1pdm09 and A/H3N2 viruses from Mauritius, 2017-2019 (Excel)


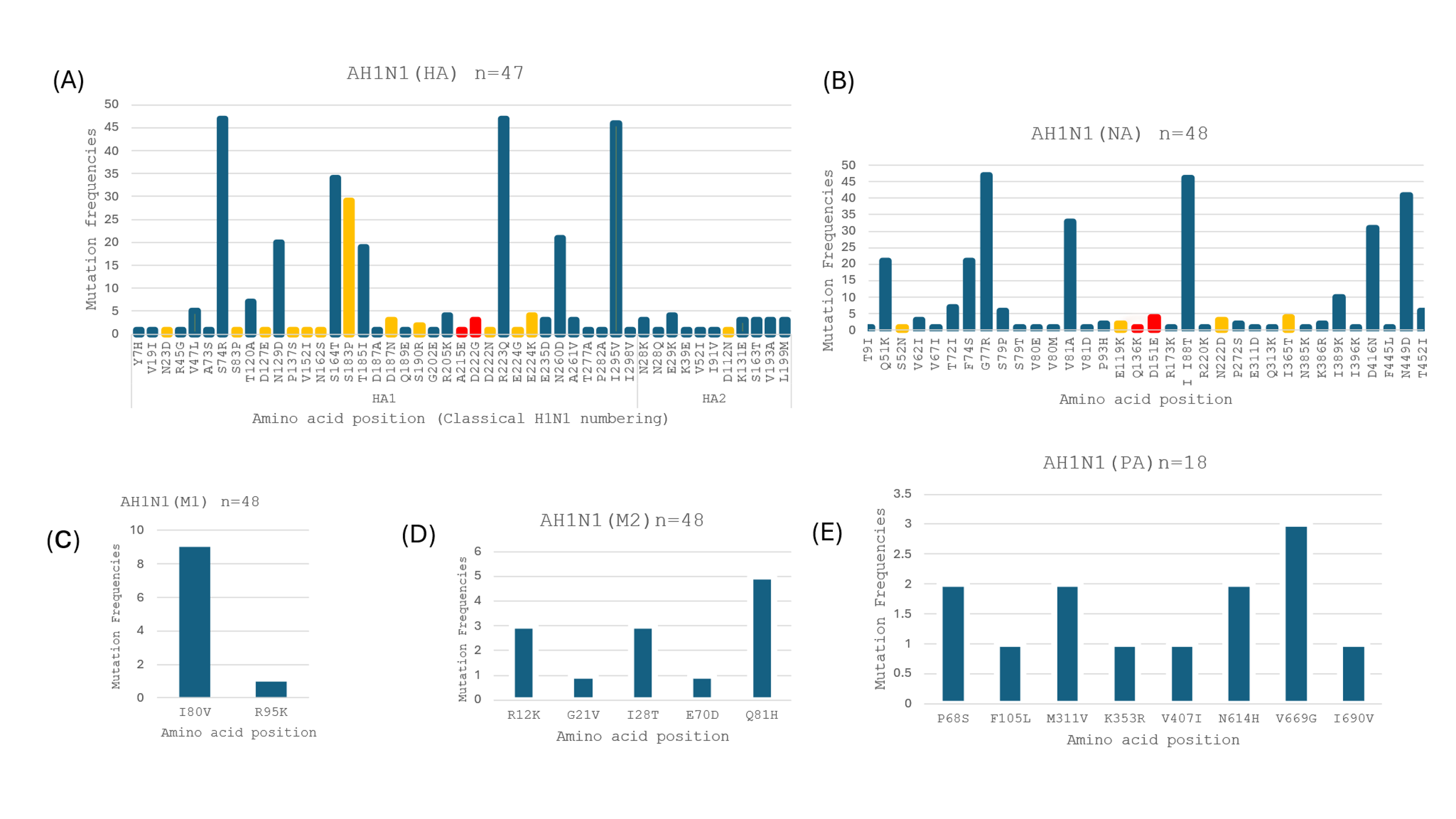


**Supplemental Figure S1.** Mutational analysis of haemagglutinin (HA), neuraminidase (NA), matrix (M) and polymerase (PA) protein of A/H1N1pdm09 (N=48) using FluSurver and using the Southern Hemisphere vaccine strain A/Michigan/45/2015 as reference. Red coloured substitutions are known to alter the virulence of the virus and cause strong drug resistance. Orange coloured substitutions occur at sites known to be involved in drug binding or alter host-cell specificity.


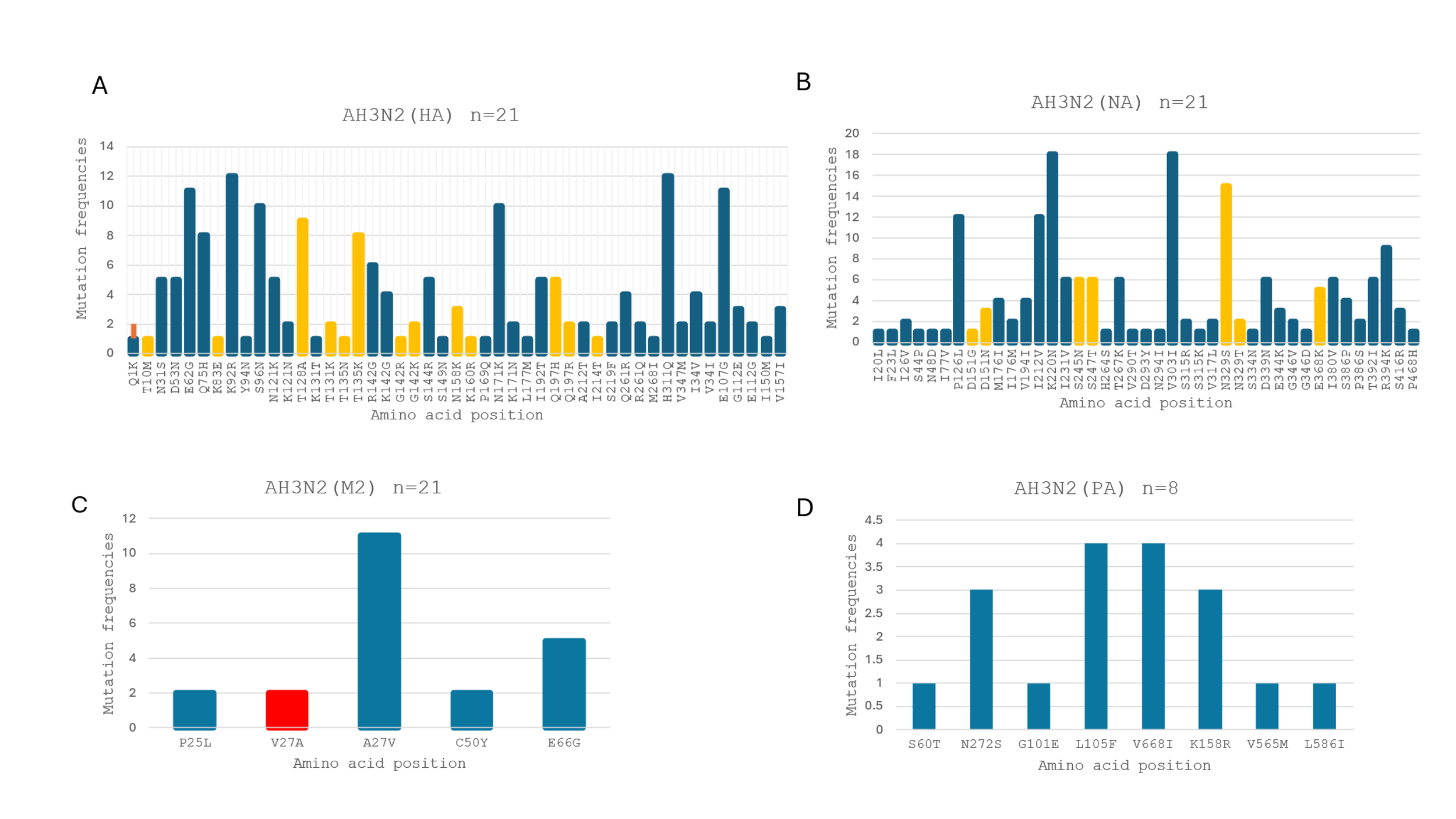


**Supplemental Figure S2.** Mutational analysis of haemagglutinin (HA), neuraminidase (NA), matrix (M) and polymerase (PA) proteins of A/H3N2 (N=21) using FluSurver and compared to the reference sequence for 2017-2019 flu seasons; A/Hong Kong/4801/2014, A/Singapore/INFIMH-16-0019/2016 and A/Switzerland/8060/2017. Red coloured substitutions are known to alter the virulence of the virus and cause strong drug resistance. Orange coloured substitutions occur at sites known to be involved in drug binding or alter host-cell specificity.
