# Supplementary material for: Molecular Evolution of Influenza A Viruses From Mauritius, 2017–2019
Source: Influenza Other Respir Viruses. 2025 May 13;19(5):e70108. doi: 10.1111/irv.70108 (PMC12074737; doi:10.1111/irv.70108)
Supplement: Supplementary file 3 — Figure S2. Mutational analysis of haemagglutinin (HA), neuraminidase (NA), matrix (M), and polymerase (PA) proteins of A/H3N2 (N = 21) using FluSurver and compared to the reference sequence for 2017–2019 flu seasons; A/Hong Kong/4801/2014, A/Singapore/INFIMH‐16‐0019/2016 and A/Switzerland/8060/2017. Red colored substitutions are known to alter the virulence of the virus and cause strong drug resistance. Orange colored substitutions occur at sites known to be involved in drug binding or alter host‐cell specificity. [file IRV-19-e70108-s003.pdf]

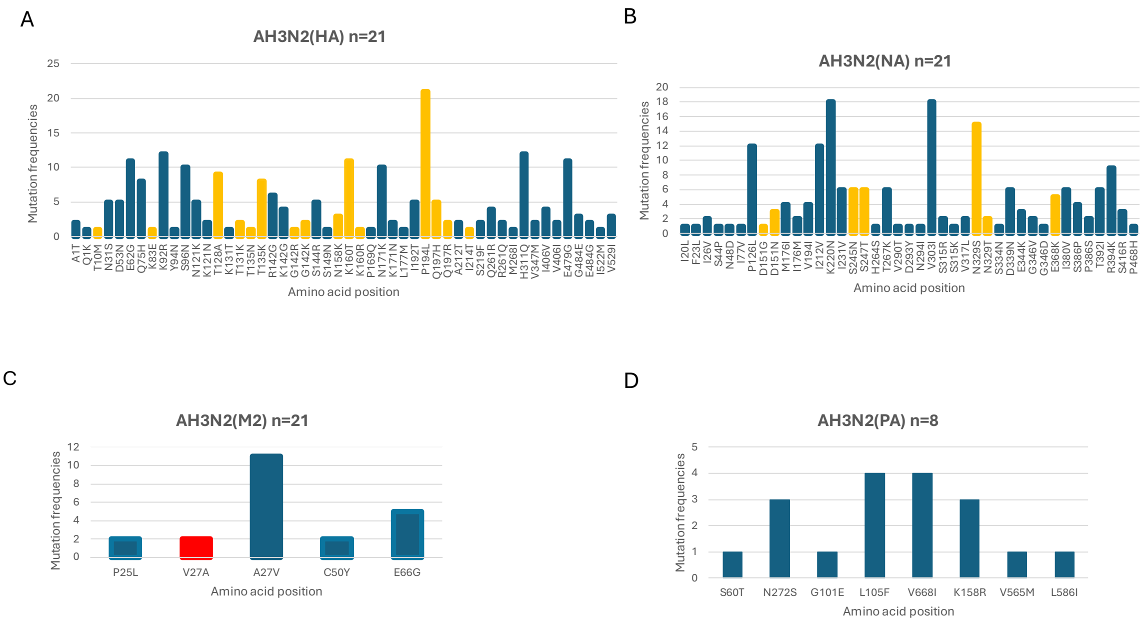

Supplemental Figure S2. Mutational analysis of haemagglutinin (HA), neuraminidase (NA), matrix (M) and polymerase (PA) proteins of A/H3N2 (N=21) using FluSurver and compared to the reference sequence for 2017-2019 flu seasons; A/Hong Kong/4801/2014, A/Singapore/INFIMH-16-0019/2016 and A/Switzerland/8060/2017. Red coloured substitutions are known to alter the virulence of the virus and cause strong drug resistance. Orange coloured substitutions occur at sites known to be involved in drug binding or alter host-cell specificity.
